# Pair-Bonding as Inclusion of Other in the Self: A Literature Review

**DOI:** 10.3389/fpsyg.2019.02399

**Published:** 2019-10-24

**Authors:** Brittany Branand, Debra Mashek, Arthur Aron

**Affiliations:** ^1^NORC at the University of Chicago, Chicago, IL, United States; ^2^Heterodox Academy, New York, NY, United States; ^3^Stony Brook University, Stony Brook, New York, NY, United States

**Keywords:** pair-bonding, self-expansion model, inclusion of other in the self, closeness, relationships

## Abstract

This article surveys scholarship on the self-expansion model principle of inclusion of other in the self (IOS) as it relates to long-term pair-bonding (i.e., enduring adult romantic monogamous relationships). First, we introduce the concept of IOS and then provide a brief overview of prior research. We then review compelling extensions and findings related to relational concepts such as perspective taking (Bernstein et al., 2015), social comparison (Thai and Lockwood, 2015), self-determination (Weinstein et al., 2016), humor (Treger et al., 2013), and pain contagion (Martire et al., 2013). Next, we explore two recent theoretical directions of the principle—the two-dimensional model of relational self-change (McIntyre et al., 2015) and the perceived inclusion of the other in the self (IOS-perceived) construct (Tomlinson and Aron, 2013). Considering these findings and their relation to pair-bonding, we propose important future directions of the IOS principle of the self-expansion model.

Pair-bonding, in the context of enduring adult romantic relationships, is the observable behavioral manifestation of an intra- and inter-psychic process of connecting with one’s partner. It is important to explore the psychological mechanisms that influence human adults to pair persistently and romantically with a specific other. One such mechanism is the self-expansion principle of inclusion of the other in the self (IOS). The self-expansion principle portends that close relationships provide opportunities to expand the self, as within relationships, each partner experiences the resources, perspectives, and identities of the other partner as to some extent one’s own. The other is to some extent “included in the self” (Aron et al., 1991). Thus, the cognitive construction of the other merges with the cognitive construction of the self, and that person’s outcomes are shared (Aron et al., 1991; Mashek and Aron, 2004). This expansion helps fulfill the human need to expand one’s efficacy. The other person informs who we are, provides new tools for our use, shapes our world view, and affects our perceived costs and benefits. The desire to include the other in the self is a dynamic motivation to pursue a pair bond; and the pair bond itself is an enduring feature of having successfully included the other in the self. The relation between the two constructs is so integrated that in essence, pair-bonding could be described as the inclusion of the other in the self and the inclusion of the other in the self is certainly an example of pair-bonding.

This paper reviews illustrations of the role and application of IOS and contributes to the understanding of the important connection between the two constructs of pair-bonding and IOS. It demonstrates that including the other in the self can in fact predict enduring adult romantic relationships and illustrates significant results of such bonding. Aron et al. (2013) conducted a comprehensive literature survey that documented previous IOS research. They included studies that highlighted both predictors and outcomes of IOS. Since the publication of the Aron et al. review in 2013, the field of IOS research has continued to broaden (Aron et al., 2013). The following discussion reviews selected studies from the 2013 review and highlights some of the work since then that has expanded and enhanced the understanding of IOS across three categories: measurement, predictors of IOS, and outcomes of IOS.

## Measurement of Inclusion of Others in the Self

To measure the closeness experienced in pair-bonded relationships, Aron et al. (1992) designed the IOS Scale. The IOS Scale features the metaphor of overlapping selves and encapsulates the construct of interconnected selves by presenting seven pairs of overlapping circles with each pair overlapping slightly more than the preceding pair. Respondents select the pair of circles that best portrays their relationship. The original validation of the IOS Scale captured aspects of both *feeling* close and *behaving* close, and correlated strongly with more complex, multi-item measures of closeness and intimacy (Aron et al., 1992).

The IOS Scale is impressively flexible and has been used cross-culturally to study diverse categories of personal relationships (e.g., Uleman et al., 2000; Dalsky et al., 2008). With its pictorial presentation, the IOS Scale presents no language barriers. Further, capitalizing on the availability of technology, a dynamic IOS Scale was created for use in Web-based data collection where a computer mouse can be used to alter the relationship between the two circles, or selves (Le et al., 2007). Although several other measures of closeness, including the implicit me-not-me task (Aron et al., 1991, Study 3), have been used successfully in much research, including to help validate the IOS Scale, the IOS Scale is the most common—and, arguably, the most face-valid—measure of inclusion. And because it is a single item, it is particularly efficient. To date, the paper that originated the IOS Scale has over 3,800 citations.

Adding to previous literature, the most recent comprehensive evaluation of the IOS Scale found it to be a psychologically meaningful and highly reliable measure of the subjective closeness of relationships for a diverse online sample of adults (Gächter et al., 2015).Offering a new strategy for assessing IOS, Castañeda et al. (2015) assessed whether Facebook profiles could be used to measure relationship closeness. They found positive associations between self-reported IOS and the couple’s Facebook overlap, which refers to how couple’s individual Facebook profiles overlap as measured in shared pictures, friends, and similar “likes.” Further, *Facebook* overlap was associated with commitment and relationship investment in ways comparable to self-reported IOS. These findings suggest that overlap in Facebook profiles can be used as an objective indicator of IOS.

Given the pair bond itself is an enduring feature of having successfully included the other in the self, measuring IOS captures depth and breadth of the pair bond.

## Predictors of Including Others in the Self

In the 2013 review, one study examining what predicts IOS found that self-disclosure was a strong mechanism for creating IOS, demonstrating experimentally that gradually increasing reciprocal self-disclosure with a stranger can create greater IOS (Aron et al., 1997). Another study found that sharing exciting activities—versus boredom—in marriage in year 7 predicted increased satisfaction in year 16, and that changes in IOS mediated this effect (Tsapelas et al., 2009). Yet another theoretically interesting approach to induce inclusion, based on Fredrickson’s (2001) broaden and build theory, found that inducing positive affect increases IOS with a close friend (Waugh and Fredrickson, 2006).

Research continues to unpack predictors of IOS. Recent insights regarding humor and attachment avoidance are introduced below.

Humor is a common interpersonal tool that has been the subject of previous research on relationships suggesting it may positively influence the trajectories of social interactions (Storey, 2003; Fraley and Aron, 2004; Wilbur and Campbell, 2011), including IOS. Treger et al. (2013) examined it in regard to how it was associated with closeness as measured by the IOS scale. In two social interaction experiments, the authors examined the association between humor and liking. In both studies, the use of humor was positively associated with liking and closeness. Perceived reciprocal liking and enjoyment of the interaction mediated the association. The findings suggest that humor is a mechanism used to establish connections with others.

Finally, a brief perspective-taking induction preceding couples’ unresolved conflict discussions was shown to interact with individual differences in attachment avoidance to influence post-conflict ratings of self-partner overlap. The authors found that the perspective-taking induction buffered the effect of partner—but not one’s own—avoidance on self-partner overlap (Bernstein et al., 2015).

The studies highlighted in this section suggest several interesting possible avenues to establish and enhance IOS—and thus pair-bonding—such as: increasing reciprocal self-disclosure, sharing in exciting activities, inducing positive affect, the use of humor, and perspective-taking training.

## Outcomes of Including Others in the Self

Outcomes of IOS were demonstrated in a number of studies included in the 2013 review: the “me/not-me” paradigm illustrated that when another person is included in the self, one’s ability to process information about the self on a particular trait is slowed to the extent that the other is dissimilar on that trait (Aron et al., 1991); confusions between self and close others were more likely than confusions between self and non-close others when recalling adjectives previously rated as describing three different targets (Mashek et al., 2003); more use of plural pronouns was correlated with more inclusion (Agnew et al., 1998); people in relationships perceived themselves as less constrained in their physical nature because they included the other’s physical attributes (Burris and Rempel, 2008); individuals processed physical pain experienced by self and a close other the same, but not with a stranger (Cheng et al., 2010); and a close other’s success was celebrated, rather than seen as threatening (Gardner et al., 2002).

More recent literature builds on this body of knowledge about the beneficial outcomes of including others in the self. For example, Weinstein et al. (2016) applied principles from self-determination theory to examine whether individual differences in self-determined motivation moderated the effects of higher self-other overlap on partner outcomes. Results showed that when self-determined individuals reported greater self-other overlap, their partners also reported receiving more positive motivational support as well as enhanced commitment. Conversely, when individuals were low in self-determination, partners did not benefit from greater self-other overlap. These results suggest that the benefits of closeness in a romantic relationship are dependent upon one’s partner approaching the relationship fully, authentically, and from their own values rather than for extrinsically motivated reasons.

A recent study (Walsh and Neff, 2018) looked at “identity fusion” and its impact on handling conflicts in pair-bonded romantic relationships. Results demonstrated that individuals who perceived greater fusion with their partner (i.e., perceived an equal blending of the personal and partner’s self in creating their unique couple identity) exhibited reduced vigilance for relationship threats and enacted more constructive coping responses to relationship conflict. On the other hand, individuals who perceived an imbalanced couple identity (i.e., perceived either their own or their partner’s identity as dominant in the couple identity) exhibited fewer of these pro-relationship behaviors. This research provides an important extension to the IOS literature by not just focusing on the amount of overlap between partners, but rather, the different ways selves can be integrated.

While experiences of closeness in romantic relationships have been found to be associated with increased levels of relational well-being and mental health (Reis et al., 2000; Reis and Aron, 2008; Holt-Lunstad et al., 2010), individuals differ in their desire for closeness in a relationship (Mashek and Sherman, 2004). To further examine that variance, a longitudinal survey of partnered individuals measured participants’ actual and ideal IOS across three time points. Results demonstrated that optimal levels of relational well-being and mental health existed when individuals had minimal discrepancies between actual and ideal IOS over time, regardless of their actual levels of IOS (Frost and Forrester, 2013). These findings suggest that closeness regulation may be an important mechanism to improve mental health and relational well-being over time, above and beyond promoting closeness itself.

Thai and Lockwood (2015) examined social comparison in the context of a romantic relationship. The authors examined whether individuals respond to comparisons involving romantic partners as they would to comparisons involving the self. Results indicated that, when reminded of their partner’s inferiority in a domain, high self-other overlap participants maintained positive global partner perceptions, whereas low overlap participants’ global perceptions were negatively affected. These results suggest that perceptions of partners remain robust when we feel a high degree of overlap with them, even when presented with specific evidence that our partners may not be perfect.

Other recent work, however, highlights potential challenges of increased self-other overlap (i.e., IOS). For example, although chronic pain has been linked to poorer psychosocial well-being in the spouse (Schwartz and Slater, 1991), the extent to which partner pain affects spouse sleep had not been researched. Martire et al. (2013) tested the hypothesis that greater daily knee pain would be associated with poorer sleep that evening for the spouse, and that the spouse’s sleep quality would be worse in couples who have a closer relationship as measured by the IOS Scale. Results indicated that greater knee pain at the end of the day was associated with spouses’ poorer overall sleep quality that night controlling for disturbances in patient sleep; this effect was stronger in couples with a high level of closeness.

In a disturbing application of IOS, Benavidez et al. (2016) examined the effect of closeness between partners in cultures of honor where women, when seen as disgracing their mate, can be violently punished. Endorsement of a “culture of honor,” where male partners’ or family members’ reputations can be tarnished by the acts of the females in the family, contributes to the belief that family honor is tied to female obedience across a variety of moral values. In this study, male participants filled out a measure of cultural honor and closeness to their wife or partner as measured by the IOS. Participants with high levels of both closeness and honor were most aggressive toward a hypothetical moral violation. In sum, within a culture of honor, the closer honor-endorsing men are to women, the more perceived violations by women are met with increased aggression.

In another direction, Slotter et al. (2014) focused on the effect of relationship dissolution on attributes that were attained through the inclusion of the other in the self. They examined factors that predicted whether individuals retain or reject attributes from their self-concept that they had initially gained during a relationship. Results indicated that individuals preserve aspects they had garnered from a former partner in their self-concepts if they had invested greater, versus lesser, psychological, mental, or physical effort to maintain those attributes; however, when these attributes actually conflict with their own previously held beliefs and attitudes, it can be confusing and lead to reduced self-concept clarity upon relationship dissolution. This research suggests that the harder one works to include another’s conflicting attributes in one’s self-concept, the more vulnerable one’s self-concept may be should the relationship end.

The studies in this section illustrate some of the benefits and challenges of a stronger pair bond. For example, those that are more bonded by including more of the other in the self are shown to practice more constructive responses to relationship conflict; to have enhanced commitment when bonded to a self-determined, authentic partner; and to maintain positive global perceptions of partners. Demonstrated challenges associated with stronger bonding include: poorer sleep when one’s partner experiences chronic pain, increased aggression within a culture of honor the closer the honor-endorsing male is to the woman, and more difficulty reconciling self-concept upon relationship dissolution.

## Recent Theoretical Directions

While the research reviewed above examines applications of IOS based on the original theoretical framework, two new theoretical directions hold promise for expanding the understanding and application of IOS as it relates to pair-bonding.

Mattingly et al. (2014) developed a theoretical framework, the two-dimensional model of relational self-change, to better understand how romantic relationships can affect an individual’s sense of self, and how those changes are related to relationship functioning. According to the model, self-concept change occurs along two independent dimensions: *direction*—whether the self-concept has lost or gained content, and *valance*—whether the self-concept content is positive or negative. These dimensions create four distinct self-change processes: two that improve self-concept—*self-expansion* (individuals gain positive traits) and *self-pruning* (individuals lose negative traits); and two that degrade self-concept—*self-contraction* (individuals lose positive traits) and *self-adulteration* (individuals gain negative traits). Mattingly et al. (2014) developed a measure of self-concept change and found that the self-concept improvement processes were associated with greater love and relationship quality, while the self-concept degradation processes were associated with more infidelity.

In a further investigation, McIntyre et al. (2015) studied how perceived relationally induced self-concept changes were associated with relationship quality, as well as relational behaviors and motivations. McIntyre et al. (2015) found that increases in self-expansion and self-pruning were associated with greater relationship satisfaction and commitment, while increases in self-contraction and self-adulteration were associated with a subsequent decrease in satisfaction and commitment. In a second study, McIntyre et al. (2015) found that self-expansion and self-pruning were positively associated with relationship maintenance behaviors such as willingness to sacrifice and forgiveness, whereas self-contraction and self-degradation were negatively associated with these outcomes and positively associated with potentially harmful relationship behaviors such as seeking revenge and attention to alternatives. The two-dimensional model suggests that the gains or losses one perceives by including another in oneself have important implications for one’s self-concept and the subsequent relationship quality they experience.

In another theoretical innovation, Tomlinson and Aron (2013) extended the IOS model to incorporate one’s perception of the extent to which the partner includes oneself in his or her self-concept by introducing a new construct—perceived inclusion of other in the self (IOS-perceived). This model posits that perceived partner satisfaction (i.e., one’s belief about how satisfied one’s partner is in the relationship) leads to perceptions of partner closeness (i.e., one’s belief about how close their partner feels to them, or IOS-perceived), which impacts one’s own closeness to the partner (IOS). IOS-perceived was measured with just a slight modification of the original IOS overlapping-circles scale: asking participants to answer as if they were their partner. In two independent studies, Tomlinson and Aron found strong support for the proposed mediational model, emphasizing the importance of measuring specific perceptions of the partner’s feelings about satisfaction and closeness.

In the first new theoretical direction discussed above, the two-dimensional model suggests that the gains or losses one perceives when pair-bonded impact one’s self-concept and relationship quality. The second illustrates the importance of one’s belief of how satisfied one’s partner is in the relationship and how close their partner feels to them in engendering closeness and encouraging reciprocal bonding.

## Conclusion and Future Directions

This article surveyed scholarship on the self-expansion model’s principle of IOS as it relates to long-term pair-bonding. The highlighted studies indicated that the constructs of pair-bonding and IOS are interrelated and complementary, as the desire to include the other in the self is a dynamic motivation to pursue a pair bond; and the pair bond itself is an enduring feature of having successfully included the other in the self. This review illustrated the utility of drawing upon IOS research when considering pair-bonding in the context of long-term adult romantic relationships.

When contemplating the future directions of the IOS principle of self-expansion as it relates to pair-bonding, a number of fertile areas present. Although the current review describes the theoretical linkage between IOS and pair-bonding, empirical research should more explicitly evaluate whether motivation for IOS is a major reason for pair-bonding. Additionally, to advance the literature regarding the association between pair-bonding and relationship dissolution, research could expand from break-up scenarios to looking at the effects of a partner’s death. This direction would prove interesting and perhaps inform bereavement counseling. It might also prove fruitful to explore IOS as a state rather than just as a trait, examining the differences between short- and long-term pair-bonded relationships. Finally, to further clarify the cognitive function of the bonding process, studies should also examine how IOS (as measured by self-report, implicit measures, and by overlap of neural systems between self and other) correlates with neural systems found for pair-bonding in animals.

## Author Contributions

BB, DM, and AA contributed to conception and design of the literature review and wrote sections of the manuscript. BB organized the literature review and wrote the first draft of the manuscript. DM and AA assisted with selection of articles. All authors contributed to manuscript revision, read and approved the submitted version.

### Conflict of Interest

The authors declare that the research was conducted in the absence of any commercial or financial relationships that could be construed as a potential conflict of interest.
